# Misleading Presentation of Multilesional Benign Pathologies in the Breast: A Diagnostic Challenge

**DOI:** 10.7759/cureus.101895

**Published:** 2026-01-20

**Authors:** A Lezith Marroquin Rodriguez, Perla Hernandez, Estefania Gomez-Charnichart, Ana Guadalupe Zapata Castillo, Javier Mohamed Reza de Leon, Luis A Solis

**Affiliations:** 1 Breast Cancer Detection and Diagnostic Center - Nuevo Leon, Hospital Regional ISSSTE, Monterrey, MEX; 2 Surgery, Hospital Regional ISSSTE, Monterrey, MEX; 3 Surgery, Universidad de Monterrey, Monterrey, MEX; 4 Radiology, Hospital Regional ISSSTE, Monterrey, MEX

**Keywords:** benign pathology, fibrocystic mastopathy, hormonal influence, keloid scar, scar formation

## Abstract

A 41-year-old female presented to our service, referred by the oncology department for evaluation of multiple nodules in the right breast. Upon questioning, the patient reported that she had sought consultation for multiple dermal lesions present for approximately 20 years in the right areolar region. However, upon examination, the physician noted palpable breast nodules and requested imaging studies for better characterization. An excisional biopsy of one of the breast nodules was subsequently performed for histopathological analysis. Because the patient’s primary concern was the longstanding dermal lesions, one of these was also excised and submitted for histopathological evaluation. The breast nodule was diagnosed as fibrocystic mastopathy, while the dermal lesion was identified as a keloid scar. Comprehensive multimodality breast imaging, including mammography, ultrasound, and contrast-enhanced MRI, is essential for accurate characterization of palpable nodules, particularly when clinical findings and patient-reported symptoms are discordant. Radiologic-pathologic correlation is especially critical in the presence of multiple lesions of differing tissue origins to ensure accurate diagnosis and appropriate management of both dermal and breast parenchymal conditions.

## Introduction

Benign breast disease is a common problem in women; epidemiological investigations indicate that more than 50% of women will develop a benign breast disorder after the age of 20 years. In this context, mastalgia is the most common presentation, followed by benign and inflammatory lesions [1]. “Fibrocystic changes” is a term used to designate a variety of clinical and histopathological changes in the female mammary gland, some of which should be regarded not so much as a disease but rather as a disruption of physiological development, maturation, and involution [2].

Between 45% and 85% of patients who attend a breast clinic do so because of this condition [3]. Fibrocystic changes are a benign alteration of the terminal duct lobular unit of the breast and are commonly observed in women of reproductive age, typically between 20 and 50 years of age [4]. The main components of the breast are susceptible to these changes during hormonal fluctuations. During the reproductive years, breast glandular tissue is closely associated with cyclic increases in plasma levels of estradiol and progesterone. Excess estrogen leads to epithelial proliferation in the lobular units of the terminal ducts and induces stromal fibrosis; this fibrosis and epithelial proliferation can obstruct ducts and acini, leading to ductal involution or the formation of cysts. When these cysts rupture, they induce adjacent fibroinflammatory stromal reactions [4]. The most common imaging modalities for evaluating these clinical findings are mammography and ultrasound [5].

Hypertrophic scars and keloids represent an abnormal healing response resulting from an exaggerated tissue repair process after injury. Keloids extend beyond the boundaries of the initial injury and exhibit a more persistent and recurrent behavior; however, the specific mechanisms remain unclear. It is believed that multiple factors influence the formation of these abnormal scars [6]. It has been suggested that sex hormones may affect the risk of keloid formation by increasing inflammation, leading to an overproduction of collagen [7]. They tend to occur in younger populations, typically aged 11 to 30 years, and are believed to result from higher epidermal renewal rates and elevated collagen production in this demographic. They can also appear spontaneously or after a previous injury, sometimes up to a year later.

Factors such as age, genetic background, race, hormones, immunity, type of wound, size, depth, location, and mechanical tension influence their development [8-9]. They are most frequently observed in individuals aged 10 to 30, with a peak incidence during puberty and early adulthood. This age-related pattern suggests a potential link between hormonal fluctuations and abnormal scar formation. These scars predominantly occur in anatomical regions subjected to mechanical stress and high skin tension, with the presternal region being the most commonly affected site. The clinical presentation typically includes firm, elevated, erythematous, or hyperpigmented lesions that may be accompanied by pruritus and pain, and they exhibit fundamental differences in their growth behavior [10].

Although fibrocystic mastopathy and other benign breast parenchymal conditions are common and well characterized, the coexistence of chronic, recurrent dermal lesions in the areolar and periareolar regions is rarely emphasized in imaging-focused literature. Such lesions may persist for decades, recur after excision, and remain clinically indolent, which contributes to underdocumentation, limited histopathological correlation, and their exclusion from formal diagnostic algorithms. This report highlights a gap in routine practice, where recurrent cutaneous breast lesions are often excised without histologic analysis, resulting in missed opportunities for definitive diagnosis and longitudinal correlation with imaging findings. Moreover, the temporal dissociation between the onset of dermal lesions (over 20 years) and the later development of palpable breast nodules underscores the need to consider dual, potentially unrelated pathologies rather than presuming a single unifying diagnosis. By documenting the imaging context, clinical course, and histopathological findings, this report contributes to the limited literature emphasizing the importance of integrating cutaneous and parenchymal breast abnormalities into a comprehensive diagnostic assessment.

## Case presentation

A 41-year-old female patient with no significant medical history was referred to the breast imaging department for a second opinion due to the presence of multiple palpable nodules limited to her right breast, which she had noticed two years prior. An excisional biopsy of one of the lesions was performed one month before presentation, with histopathological results consistent with fibrocystic mastopathy. Her primary reason for consultation was the presence of protruding dermal lesions in the same breast, which had begun approximately 20 years earlier; these lesions appeared spontaneously and remained confined to the areolar region, with no associated symptoms or inflammatory signs. Notably, the patient reported no similar cutaneous lesions elsewhere on her body.

**Figure 1:**
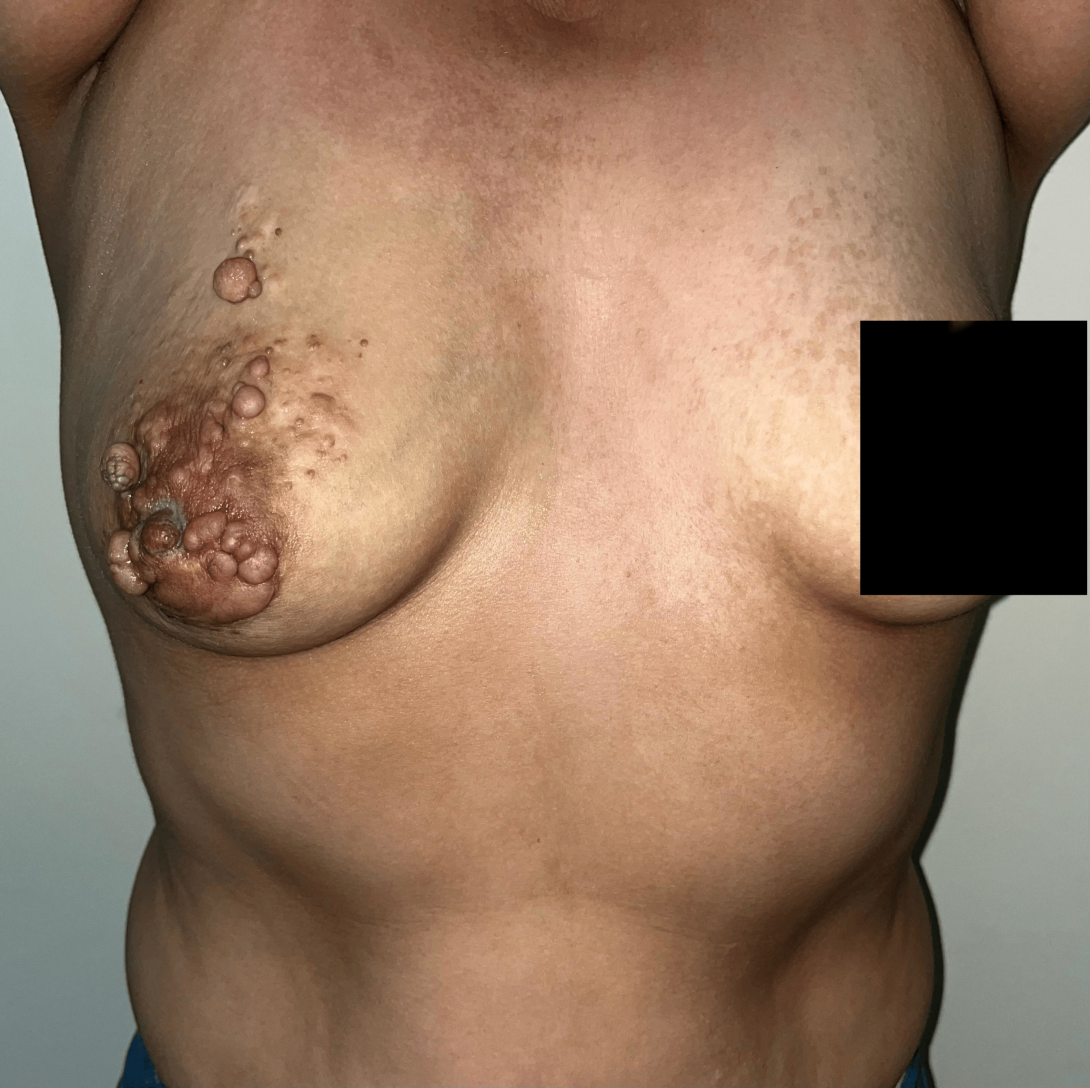
Patient image Multiple dermal lesions were observed in the right areolar region, with a sessile base, soft consistency, and brown color

The excision of these lesions was the only treatment she had received during previous medical evaluations, and recurrence was observed after each procedure. She reported that the lesions had been surgically removed on three occasions; however, none of the excised specimens were submitted for histopathological examination. The patient denied pain, nipple discharge, skin retraction, or systemic symptoms throughout the course of the disease. At the time of presentation, there was no personal or family history of breast malignancy or dermatologic disorders, and she was not receiving hormonal therapy or other long-term medications. She had never had any hormonal imbalances or menstrual abnormalities. Figure 1 shows an image of the affected breast.

**Figure 2:**
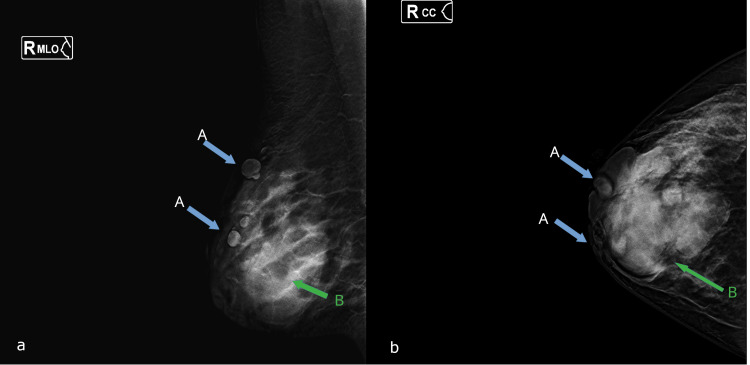
Mammography findings Standard mammographic views of the right breast, including mediolateral oblique (a) and craniocaudal (b) projections, demonstrate at least two isodense, oval-shaped nodules with well-defined margins, associated with punctate calcifications within the breast parenchyma. At the skin level, approximately four isodense, rounded, and well-circumscribed nodules are identified, predominantly in the periareolar region (blue arrows, A). The largest superficial lesion is located in the upper outer quadrant (green arrow, B)

Mammography findings are presented in Figure 2.

**Figure 3:**
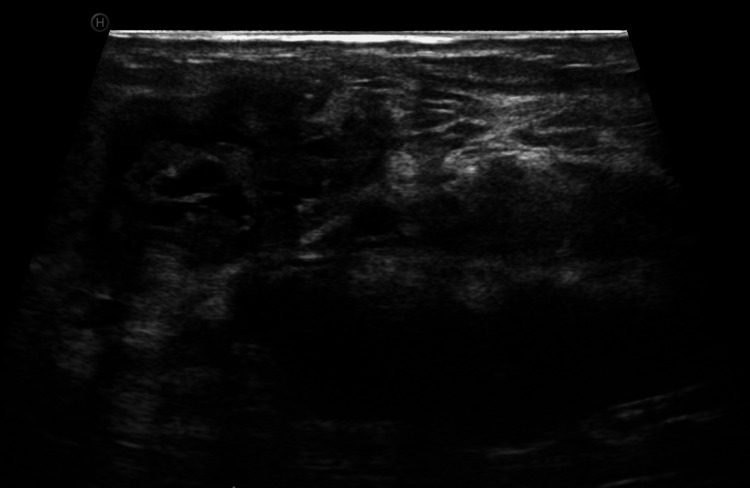
Ultrasound findings Gray-scale ultrasound image demonstrating multiple oval, well-circumscribed nodules with heterogeneous echogenicity, containing cystic components

 Ultrasound findings are illustrated in Figure 3.

**Figure 4:**
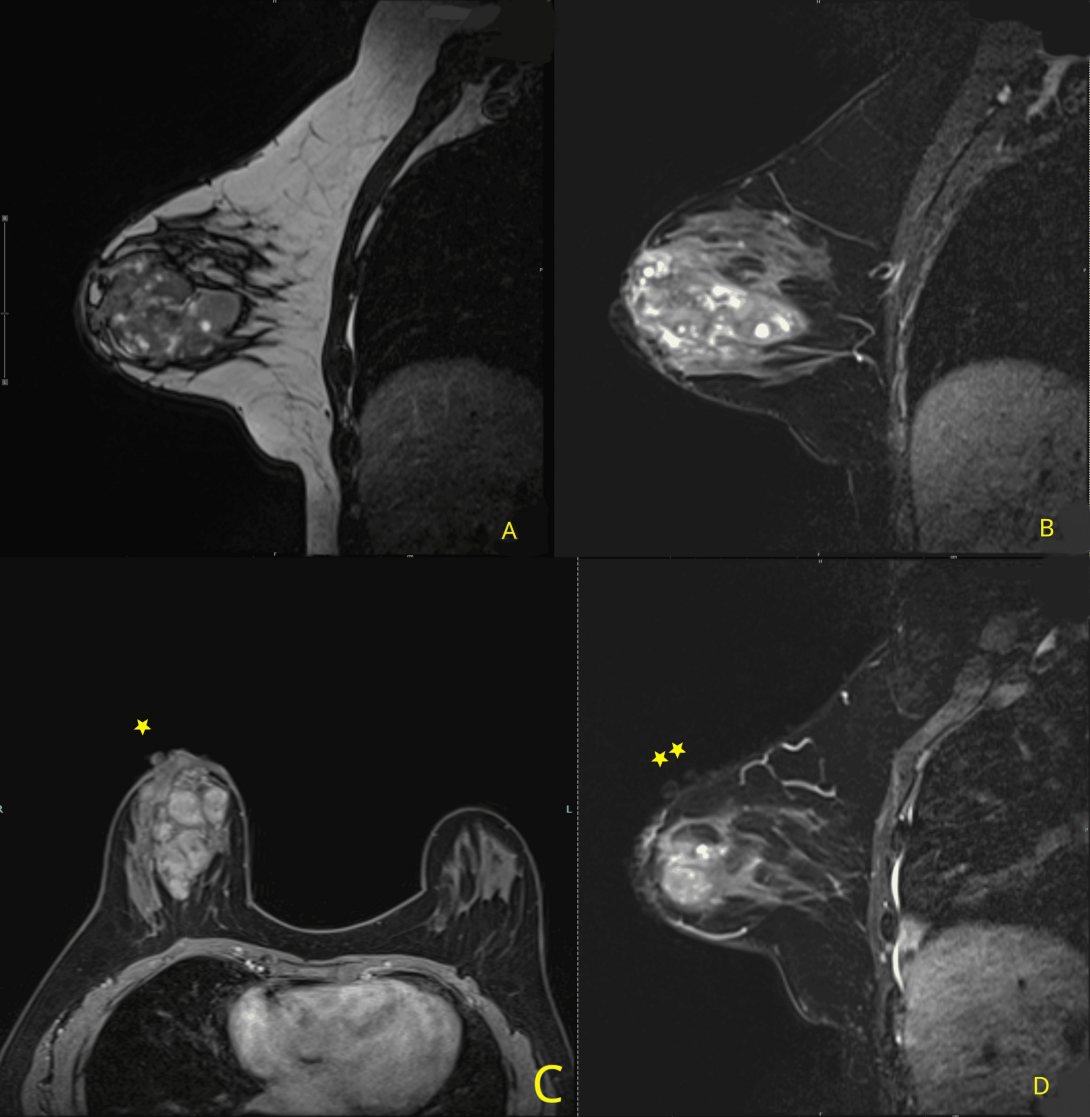
MRI findings (A) Sagittal T1-weighted image and (B) Sagittal T2-weighted image show that the right breast parenchyma presents a heterogeneous nodule with cystic areas inside. (C) Axial T1-weighted fat sat C+ (gadolinium) shows no enhancement, neither the dermal lesions (yellow stars) nor the parenchymal nodules, post-contrast administration. (D) Sagittal T2-weighted image shows the dermal lesions (yellow stars) of the same intensity, following the path and contour of the skin MRI: magnetic resonance imaging

MRI findings are shown in Figure 4.

**Figure 5:**
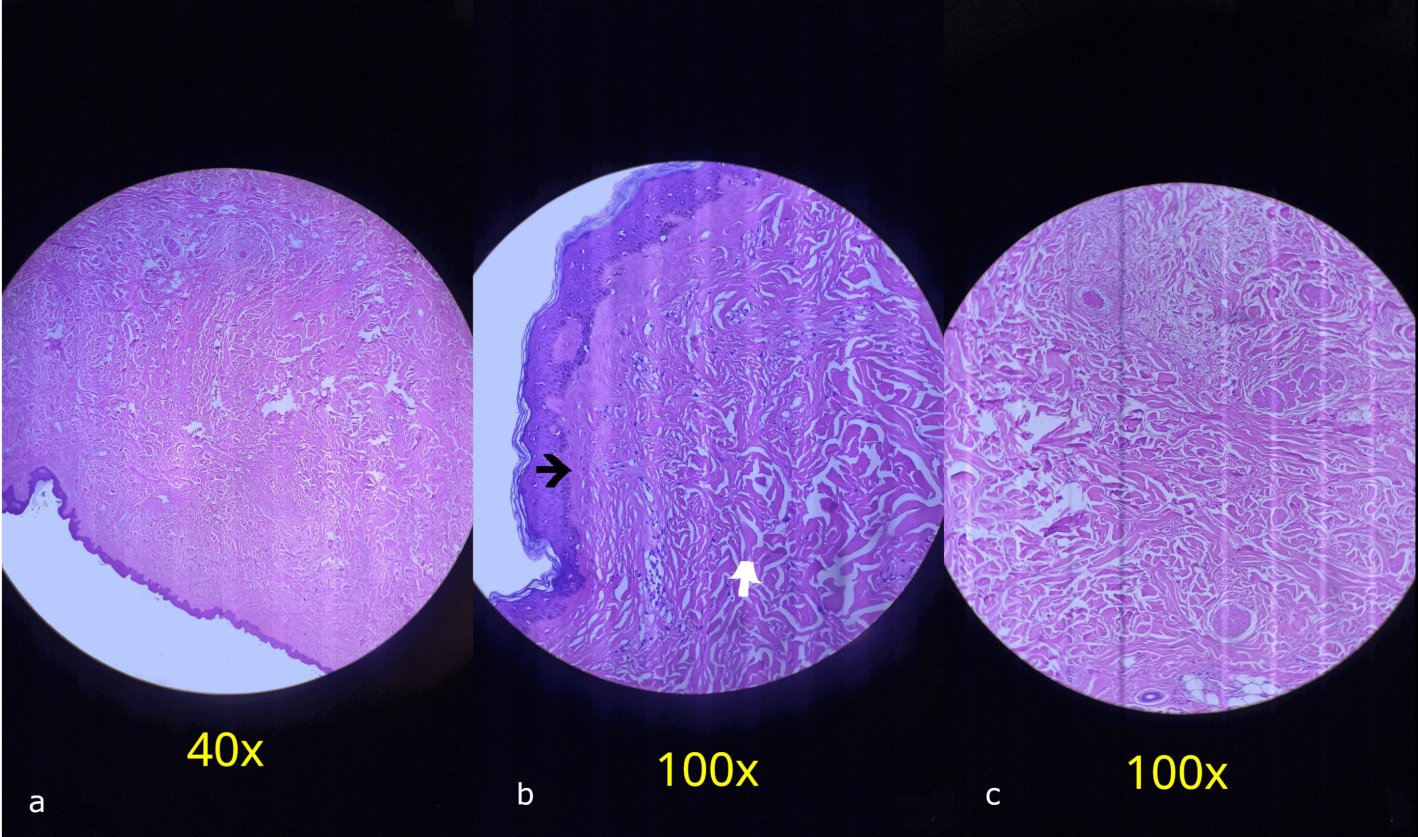
Microscopy findings (a) Low-power micrograph (40×) showing intact epidermis overlying the lesion. (b) Higher magnification micrograph (100×) demonstrating replacement of the papillary dermis by a hyalinized collagen band (black arrow) and disorganized proliferation of thick collagen bundles within the reticular dermis, associated with scant chronic inflammatory infiltrate predominantly composed of lymphocytes (white arrow). (c) Additional high-power view (100×) confirming dense, irregular collagen deposition without evidence of invasion into underlying tissues. Immunohistochemistry was not considered necessary

Microscopy findings are depicted in Figure 5.

## Discussion

This report describes a 41-year-old female patient with unilateral fibrocystic breast disease associated with long-standing, recurrent skin lesions confined to the areolar region of the same breast. This presentation is unusual and clinically challenging. Fibrocystic breast disease is the most common benign breast condition in women of reproductive age, affecting up to 50% of women clinically and up to 90% histologically [1,2]. However, its co-occurrence with persistent, recurrent cutaneous lesions limited to a single breast is rarely reported in the literature. After the age of 30, approximately half of women develop fibrocystic breast disease, a condition believed to be related to hormonal imbalances, particularly increased estrogen stimulation and decreased progesterone levels [2]. These findings support the classification of fibrocystic breast disease as a hormone-dependent condition.

The most distinctive feature of this report is the presence of protuberant, recurrent skin lesions confined exclusively to the areolar region. These lesions appeared spontaneously, were managed solely by surgical excision, and recurred after each intervention. Notably, none of the previously excised lesions underwent histopathological evaluation, which represents a critical limitation in prior management. Recurrent cutaneous lesions of the breast require thorough pathological assessment, as clinical appearance alone is insufficient to distinguish benign from fibroproliferative or neoplastic conditions [5].

Similarly, hormonal factors have been implicated in keloid pathogenesis. Sex hormones are believed to stimulate inflammatory responses that contribute to excessive collagen production, thereby increasing the likelihood of keloid development [7]. The coexistence of fibrocystic breast disease and recurrent areolar fibroproliferative lesions may be incidental; however, hormonal influences could represent a shared contributing factor. The patient experienced recurrent skin lesions beginning at age 20, without an identifiable triggering factor or prior trauma, and later developed palpable nodules within the breast parenchyma at age 39. Fibrocystic breast changes typically occur between the ages of 20 and 50, while keloid formation is more prevalent between the ages of 10 and 30. The temporal overlap observed in this patient, along with the recurrent nature of both conditions, suggests a possible shared hormonal influence in their development.

In addition to hormonal influences, mechanical factors play an important role in the formation of hypertrophic scars and keloids. These lesions occur more frequently in anatomical regions exposed to high skin tension and repetitive mechanical stress, such as the shoulders, anterior chest, and joints [10]. Furthermore, repeated surgical excisions without adjuvant therapy may perpetuate dysregulated wound healing, further increasing the risk of recurrence.

Careful correlation between physical examination and imaging planes is essential to distinguish dermal, subdermal, and parenchymal abnormalities. This case highlights several clinically relevant lessons. First, all recurrent dermal lesions of the breast should undergo histopathological evaluation to establish an accurate diagnosis and guide appropriate management. Second, surgical excision alone is insufficient for suspected keloid or fibroproliferative lesions, as recurrence rates of 50-70% may occur without adjuvant therapy [10]. Current evidence supports multimodal approaches, including intralesional corticosteroids, silicone-based therapies, pressure therapy, and laser treatment, to reduce recurrence and improve outcomes [8-10].

Finally, multidisciplinary collaboration between breast imaging, dermatology, pathology, and surgery is essential when parenchymal and cutaneous breast pathologies coexist. All excised breast or areolar lesions, regardless of presumed benignity, should be submitted for histopathological analysis, as recurrence alone warrants diagnostic clarification. Ultimately, this presentation underscores that breast imaging should not be considered in isolation from dermatologic processes. Heightened awareness of atypical, long-standing cutaneous lesions can improve diagnostic accuracy, prevent repeated non-diagnostic interventions, and promote a more comprehensive, patient-centered approach to breast evaluation.

## Conclusions

This report suggests a possible shared hormonal influence underlying an unusual but benign presentation. Although the patient initially sought care for aesthetic concerns, the subsequent development of a palpable nodule prompted appropriate diagnostic evaluation, which confirmed the presence of benign disease. Given the benign characteristics of the findings across imaging modalities and the concordance between imaging and histopathological results, a Breast Imaging Reporting and Data System (BI-RADS) 2 classification with routine annual surveillance is justified in the absence of clinical progression. The coexistence of unilateral fibrocystic breast disease with long-standing, recurrent areolar skin lesions represents a rare and diagnostically challenging association. This report underscores the necessity of histopathological evaluation of recurrent breast skin lesions and highlights the value of a multidisciplinary, multimodal approach to prevent repeated non-diagnostic interventions.
